# Binding Specificities of the Telomere Phage ϕKO2 Prophage Repressor CB and Lytic Repressor Cro

**DOI:** 10.3390/v8080213

**Published:** 2016-08-03

**Authors:** Jens Andre Hammerl, Claudia Jäckel, Erich Lanka, Nicole Roschanski, Stefan Hertwig

**Affiliations:** 1 Bundesinstitut für Risikobewertung (Federal Institute for Risk Assessment), Department of Biological Safety, Diedersdorfer Weg 1, D-12277 Berlin, Germany; jens-andre.hammerl@bfr.bund.de (J.A.H.); claudia.jaeckel@bfr.bund.de (C.J.); 2 Max-Planck-Institut für Molekulare Genetik, Ihnestraße 63-73, D-14195 Berlin, Germany; lanka@molgen.mpg.de; 3 Institute of Animal Hygiene and Environmental Health, Free University Berlin, Robert-von-Ostertag-Str. 7-13, D-14163 Berlin, Germany; nicole.roschanski@fu-berlin.de

**Keywords:** telomere phages ϕKO2, N15, PY54, genetic switch, immunity region, prophage repressor CB, lytic repressor Cro, DNA binding

## Abstract

Temperate bacteriophages possess a genetic switch which regulates the lytic and lysogenic cycle. The genomes of the temperate telomere phages N15, PY54, and ϕKO2 harbor a primary immunity region (*immB*) comprising genes for the prophage repressor (*cI* or *cB*), the lytic repressor (*cro*) and a putative antiterminator (*q*). The roles of these products are thought to be similar to those of the lambda proteins CI (CI prophage repressor), Cro (Cro repressor), and Q (antiterminator Q), respectively. Moreover, the gene order and the location of several operator sites in the prototype telomere phage N15 and in ϕKO2 are reminiscent of lambda-like phages. We determined binding sites of the ϕKO2 prophage repressor CB and lytic repressor Cro on the ϕKO2 genome in detail by electrophoretic mobility shift assay (EMSA) studies. Unexpectedly, ϕKO2 CB and Cro revealed different binding specificities. CB was bound to three *O_R_* operators in the intergenic region between *cB* and *cro*, two *O_L_* operators between *cB* and the replication gene *repA* and even to operators of N15. Cro bound exclusively to the 16 bp operator site *O_R_3* upstream of the ϕKO2 prophage repressor gene. The ϕKO2 genes *cB* and *cro* are regulated by several strong promoters overlapping with the *O_R_* operators. The data suggest that Cro represses *cB* transcription but not its own synthesis, as already reported for PY54 Cro. Thus, not only PY54, but also phage ϕKO2 possesses a genetic switch that diverges significantly from the switch of lambda-like phages.

## 1. Introduction

Temperate phages can choose between two life cycles, the lytic and lysogenic cycles. During the lysogenic cycle most temperate phages are integrated into the bacterial chromosome. Within the lambdoid phage group, the temperate phages N15, PY54, and ϕKO2 isolated from *Escherichia coli (E. coli)*, *Yersinia enterocolitica* (*Y. enterocolitica*), and *Klebsiella oxytoca (K. oxytoca)*, respectively, belong to a particular subgroup because their prophages are not integrated into the bacterial chromosome, but replicate as linear plasmids with covalently closed hairpin ends (telomeres) [1,2,3]. Telomere phages have also been found in *Vibrio parahaemolyticus* strains (VP58.5, VP882, and vB_VpaM_MAR) and in *Halomonas aquamarina* (HAP-1) [4,5,6,7]. It is notable that enterobacterial telomere phages are members of the family *Siphoviridae*, whereas all hitherto described marine telomere phages belong to the *Myoviridae*. However, even though there are only partial protein similarities between enterobacterial and marine telomere phages, these phages share a similar genome organization [8].

Like other temperate phages, telomere phages possess genes providing the genetic switch between the lytic and the lysogenic cycle. Sequence analyses suggested that telomere phages possess a primary immunity region (*immB*) which is comparable to the immunity region of lambda-like phages but exhibits a simpler arrangement [9] (Figure S1). In the enterobacterial telomere phages, *immB* encodes products related to the prophage repressor CB (prophage repressor CI in PY54), lytic repressor Cro, and a putative antiterminator Q as well as operator sites (three in N15 and ϕKO2, and one in PY54) located between *cB* and *cro* [8,10]. Due to the close relationship between the *immB* regions of N15 and ϕKO2, the same repressor target specificity for these phages has been suggested [1,11]. Indeed, using in vivo assays it has been shown that the genes for the prophage repressor, lytic repressor and Q of ϕKO2 influenced lysis and lysogeny of N15 in the same way as their counterparts of N15 [2,12]. By contrast, none of the investigated PY54 genes affected the N15 life cycles but exerted specific activities in *Yersinia* [8,10]. The N15 prophage repressor has already been studied in detail. Similar to lambda, binding was observed to the three operators situated between *cB* and *cro*. In addition, two operators (*O_L_*) overlapping with the predicted promoter of the plasmid replication gene *repA* were bound. N15 CB was, therefore, suggested to be implicated in both the regulatory circuitry of phage propagation and the control of plasmid replication [9]. Contrary to the prophage repressor, there is a lack of information about the activity of the Cro protein. Dubrava et al. studied the structure and dimerization of the N15 Cro protein in comparison to lambda and P22 Cro [11]. The authors showed that the subunit fold of N15 Cro contains five alpha-helices and is very similar to the fold of P22 Cro, while dimerization of N15 Cro is much stronger and comparable to that of lambda Cro. Therefore, N15 Cro may represent a new category of Cro proteins, the all-alpha helical dimers, to which the ϕKO2 lytic repressor most likely belongs as well. Similarities to lambdoid phages enabled Hall et al. to predict amino acid residues of the recognition helices of N15 Cro and base pairs within operator half-sites probably involved in binding to the repressor [13]. However, experimental data on this issue are still missing. Lytic repressor activity of the telomere phages has yet been demonstrated only for Cro of PY54. This protein revealed a high binding specificity for a single site (*O_R_3*) adjacent to the prophage repressor gene but did not bind to closely-related N15 and ϕKO2 operators. Thus, PY54 Cro apparently suppresses *cB* transcription, but not its own synthesis, suggesting that the genetic switch of this phage diverges significantly from those of N15 and ϕKO2 [10].

In this work we determined binding specificities of the ϕKO2 CB and Cro repressors. We show that the prophage repressor CB binds to the three *O_R_* and two *O_L_* operators on the ϕKO2 genome. By contrast, the ϕKO2 Cro lytic repressor is highly specific with respect to its DNA target. It was exclusively bound to the ϕKO2 *O_R_3* operator site upstream of *cB*. The data demonstrate that the *immB* region of ϕKO2 is only structurally, but not functionally, similar to the genetic switch of lambda.

## 2. Materials and Methods

### 2.1. Bacterial Strains, Phages, Plasmids, and Growth Conditions

Detailed information on bacterial strains and plasmids used in this study is given in Table S1. The *E. coli* strains GeneHogs (Invitrogen, Karlsruhe, Germany) and DH5α were used for standard cloning procedures [14]. Overexpression of the ϕKO2 *cB* and *cro* genes was performed in *E. coli* SCS1 (Stratagene, Amsterdam, Netherlands) and *E. coli* UT5600 (Biolabs, Frankfurt am Main, Germany). Bacteria (*K. oxytoca* and *E. coli*) were cultivated in lysogeny broth (LB) at 37 °C [15,16]. Solid media contained 1.8% (w/v) bacto agar (Oxoid, Dassel, Germany; Agar No. 1).

### 2.2. Overproduction and Purification of the Repressor Proteins

The ϕKO2 *cB* and *cro* genes were amplified by polymerase-chain reaction (PCR) using purified phage DNA as a template. Forward and reverse primers contained restriction sites for NdeI and HindIII (Fermentas, St. Leon-Rot, Germany), respectively. After digestion, the genes were inserted into the corresponding sites of pMS470Δ8cat [4,17] yielding pJH541-2 (ϕKO2 *cB*), and pJH542-2 (ϕKO2 *cro*) (Table S1). Transformants containing pJH541-2 or pJH542-2 were grown under shaking (180–220 rpm) at 37 °C in Super Optimal broth with Catabolite repression (SOC) medium (Sigma-Aldrich, Darmstadt, Germany) supplemented with 3-(*N*-morpholino) propanesulfonic acid (MOPS, Sigma-Aldrich) sodium salt (pH 8.0, 25 mM). Gene expression was induced by supplementing isopropyl β-d-1-thiogalactopyranoside (IPTG, Sigma-Aldrich) to a final concentration of 1 mM when optical density at 588 nm (OD_588_) reached 0.5. After a cultivation of 4 h, bacteria were harvested by centrifugation (4000 × *g*, 10 min) and prepared for chromatographic purification of the repressors as previously described [10].

Purification of CB and Cro was achieved by four and five steps, respectively. Fraction I containing the eluted ammonium precipitate in buffer A (20 mM Tris-HCl (pH 7.6), 1 mM 1,4-dithiothreitol (DTT, Sigma-Aldrich), 0.1 mM ethylenediaminetetraacetic acid (EDTA, Sigma-Aldrich), 10% (w/v) glycerol) with 125 mM NaCl was loaded onto an equilibrated Heparin-Sepharose CL-6B column (Sigma-Aldrich) (2.6 × 15 cm). Proteins were eluted with a 500 mL linear gradient of 100–750 mM NaCl in buffer A. Repressor peak fractions were pooled (fraction II) and subsequently applied onto different anion and cation exchanger (Heparin sepharose, DEAE sephacel, CM sepharose fast flow, and Hydroxylapatite bio gel HT, Sigma-Aldrich). The peak fraction of the final purification step was concentrated by dialysis against 20% (w/v) polyethylene glycol (PEG) 20,000 in buffer A, dialyzed against 50% glycerol in buffer A and stored at −20 °C. 

### 2.3. Sodium Dodecyl Sulfate-Polyacrylamide Gel Electrophoresis (SDS-PAGE) and Mass Spectrometric Analysis

SDS-PAGE was performed on 12.5% (w/v) polyacrylamide (PAA) gels at 15 °C according to the standard procedure [10,18]. For verification, protein bands of interest were excised from PAA gels, purified and investigated by mass spectrometry (Voyager-DE MALDI-TOF system, Applied Biosystems, Foster City, CA, USA) as previously described [19]. The obtained data were analyzed using the Mascot webserver database [20].

### 2.4. Electrophoretic Mobility Shift Assays (EMSA)

If not otherwise indicated, repressor binding to DNA targets was investigated by the use of recombinant plasmids (Table S1). The respective DNA regions of the ϕKO2, N15, and PY54 genomes were amplified by PCR with forward and reverse primers containing restriction sites for BamHI and HindIII (Fermentas), respectively. Digested PCR products were inserted into pBR329 [21] and confirmed by Sanger sequencing to avoid multiple insertions.

For CB and Cro repressor binding studies, 100 ng ClaI and HincII (Fermentas) digested plasmid DNA of the target constructs were incubated with increasing amounts of the repressor protein (0.01, 0.06, and 0.125 μM) in binding buffer (10 mM Tris-HCl (pH 7.6), 10% glycerol, 0.1 mM EDTA, 0.3 µg/µL bovine serum albumin (BSA, Sigma-Aldrich), 1 mM DTT (Sigma-Aldrich)) for 30 min at 30 °C. Repressor/DNA complexes were analyzed on 1.75% PAA gels in Tris-borate EDTA (TBE) buffer (89 mM Tris (pH 8.9), 89 mM borate, 1 mM EDTA) at 20 °C. Electrophoresis was carried out under non-denaturing conditions at 6 V/cm for 3.5 h. PAA gels were stained with SYBR-green (Cambrex BioScience, Rockland, ME, USA) for 30 min and analyzed with the Alpha Digi Doc software (Biometra, Göttingen, Germany).

Dissociation constants (K_D_) of repressor binding to different target regions were determined by gel retardation assays using synthetic double-stranded DNA fragments [22]. For this purpose, 100 ng template DNA were 5′-end labelled with [γ-^32^P] ATP (Hartmann Analytics, Braunschweig, Germany) using T4 polynucleotide kinase (Fermentas). After purification of the labelled DNA fragments on MicroSpin G-25 columns (GE Healthcare, Piscataway, PA, USA), a DNA concentration of 1 nM per reaction was used for binding studies with increasing protein concentrations. After 30 min of incubation at 37 °C, samples were analyzed on 3.5% non-denaturing PAA-gels at 6 V/cm. Vacuum dried gels were examined on a PhosphorImager (Molecular Dynamics, Sunnyvale, CA, USA). Band intensities were determined using ImageQuant v3.0 (Molecular Dynamics). Dissociation constants were calculated using the SIMFIT program (W.G. Bardsley, University of Manchester, UK, http://www.simfit.org.uk).

### 2.5. In Silico Analyses

Sequence analyses and alignments were carried out using the DS Gene software (Accelrys Inc., San Diego, CA, USA). In silico promoter identification was performed by using BPROM (Softberry, Mount Kisco, NY, USA).

### 2.6. Analysis of Promoter Activity

Promoter activity was studied using the vector pKKlux (Ap^r^), which is a promoter reporter derivative of pKK232-8 carrying the promoterless *luxAB* genes of the bioluminescent bacterium *Vibrio harveyi* [20,23]. To study the activity of promoters within the intergenic region between *cB* and *cro*, the respective DNA fragments were amplified by PCR with forward and reverse primers containing restriction sites for SmaI and XbaI (Fermentas), respectively. Fragments additionally containing *cB* or *cro* were produced in the same way. The PCR products were digested with SmaI and XbaI and inserted into the corresponding sites of pKKlux, approximately 130 bp upstream of the *lux* genes. A suitable ribosome binding site (AGGAAA) is located just in front of *luxA*. Upon transformation of *E. coli*, recombinant plasmids were verified by Sanger sequencing. Luminescence measurements were carried out in *E. coli* strain DH5α [10,23]. Luminescence from three independent experiments done in triplicate was measured at 28 °C for 10 s in a Microlumat LB96P (EG and G Berthold, Bad Wildbach, Germany) and compared with the promoterless pKKlux vector and pKKL700lux, a plasmid carrying the strong ST-LS1 promoter of *Solanum tuberosum* [23].

## 3. Results and Discussion

### 3.1. ϕKO2 and N15 Possess Closely Related *immB* Regions

**Figure 1:**
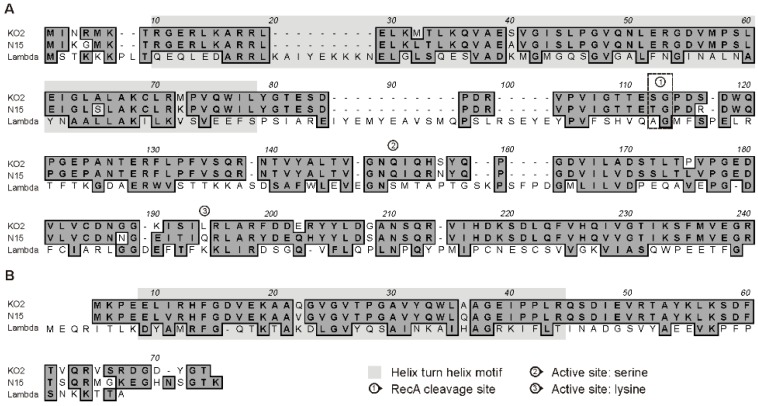
Sequence alignments of the prophage repressor proteins CB (prophage repressor CI) (**A**) and the lytic repressor Cro (**B**). Similar residues at corresponding positions are shown in dark grey. Domains for alpha helices within the N15/ϕKO2 repressors and active sites of the lambda repressors are indicated.

Sequence analyses of the genomes of enterobacterial telomere phages disclosed a similarly organized *immB* region comprising genes potentially coding for a prophage repressor, Cro repressor and antiterminator Q [10]. However, while the repressor proteins of phage PY54 are only distantly related (less than 40% identity) to their counterparts in ϕKO2 and N15, the repressors of these two phages are highly homologous exhibiting sequence identities of 89% (CB) and 88% (Cro) [8]. Overall similarities to CI and Cro of lambda are low but a helix-turn-helix motif has been identified in all repressors (Figure 1). By contrast, a putative RecA cleavage site and two active sites comprising essential residues (serine and lysine) that are strictly conserved among self-cleaving repressors, including the bacterial repressor LexA [17], were not detected in CB of ϕKO2 and N15 (Figure 1). Unlike lambda which contains six 17 bp operators for repressor binding, ϕKO2 and N15 possess five 16 bp operator sites, three of which (*O_R_1*, *O_R_2* and *O_R_3*) are embedded in the intergenic region between *cB* and *cro*, whereas two *O_L_* operators (*O_L_1* and *O_L_2*) are located upstream from the replication gene *repA* [10]*.* The operators of ϕKO2 and N15 are very similar or even identical (*O_R_3*), all of them possess a T-A-(N)_10_-T-A motif [10]. Binding to all five operator sites has been demonstrated for the N15 prophage repressor [9]. No experiments have yet been performed with N15 Cro and with the ϕKO2 repressors, even though similar binding specificities have been predicted for these proteins [1]. We, therefore, studied the binding specificities of the ϕKO2 repressors in detail. 

### 3.2. CB and Cro Do Not Bind to the Same Operators

**Figure 2:**
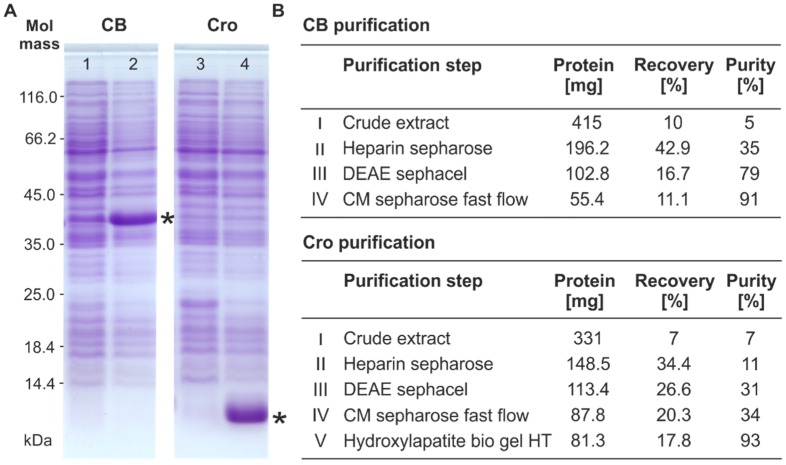
Overproduction and purification of the ϕKO2 CB and Cro repressor. (**A**) Sodium dodecyl sulfate-polyacrylamide gel electrophoresis (SDS-PAGE) of crude extracts of *Escherichia coli* strains containing the repressor genes. Lanes 1 and 3 show protein profiles of non-induced controls, lanes 2 and 4 show protein profiles after isopropyl β-d-1-thiogalactopyranoside (IPTG) induction. Repressor bands are indicated by asterisks; (**B**) Chromatographical purification of the ϕKO2 CB (upper panel) and Cro repressor (lower panel). Detailed information on the recovery and purity of the repressors at the different purification steps are given. DEAE, Diethylaminoethyl–Sephacel^®^; CM, Carboxymethyl Sepharose; HT, Hydrated.

For the isolation of the ϕKO2 repressors, the genes *cB* and *cro* were amplified by PCR and introduced into *E. coli* laboratory strains using the expression vector pMS470Δ8cat (pJH541-2 [cB]/pJH542-2 [cro]; see Materials and Methods). Gene expression was induced by the addition of IPTG. Upon induction, proteins of approximately 37 kDa (CB) and 8 kDa (Cro) were produced, which is in good agreement with the molecular masses of the proteins deduced from sequence data. The repressors were purified close to electrophoretic homogeneity by a five step purification procedure (Figure 2). The authenticity of the proteins was proved by Matrix-Assisted Laser Desorption/Ionization-Time of Flight (MALDI-TOF) mass spectrometry (data not shown).

**Figure 3:**
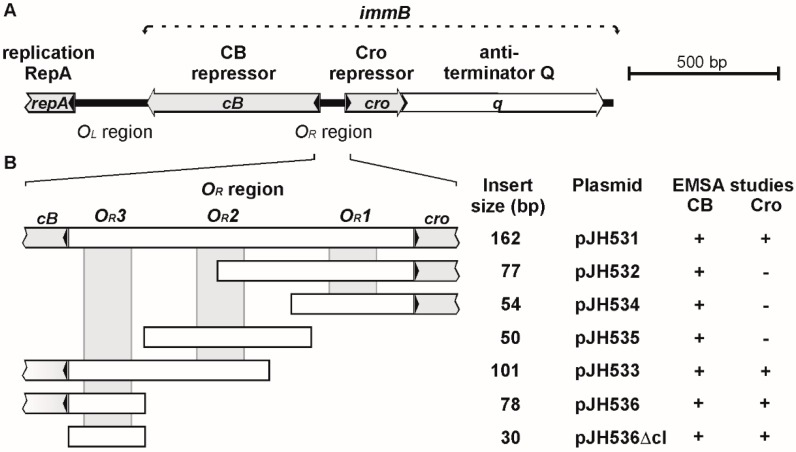
Determination of the ϕKO2 CB and Cro binding sites. (**A**) Schematic overview of the primary immunity region (*immB*) of ϕKO2; (**B**) binding assays were performed by electrophoretic mobility shift assay (EMSA) using recombinant plasmids. On the right, the sizes of the respective substrates and the results of the repressor binding tests are presented (+, band shift; -, no band shift).

Initial binding assays (electrophoretic mobility shift assay, EMSA) were performed with cloned DNA fragments containing at least one *O_R_* operator site. All tested fragments showed binding to the ϕKO2 prophage repressor CB (Figure 3) whereas only fragments harboring the *O_R_3* site were bound by Cro. Thus, CB and Cro diverge in terms of their binding properties. While the ϕKO2 prophage repressor is similar in this respect to N15 CB, the high binding specificity of Cro resembles that of the PY54 lytic repressor [10]. Nevertheless, the close relationship of the ϕKO2 and N15 Cro proteins and operators suggest that also the lytic repressors of these two phages have the same binding properties [1]. On the other hand, although the exact binding sites of the PY54 prophage repressor are not known, the presence of a single operator in the intergenic region between *cI* and *cro* suggests a different mechanism of gene regulation in this phage.

**Figure 4:**
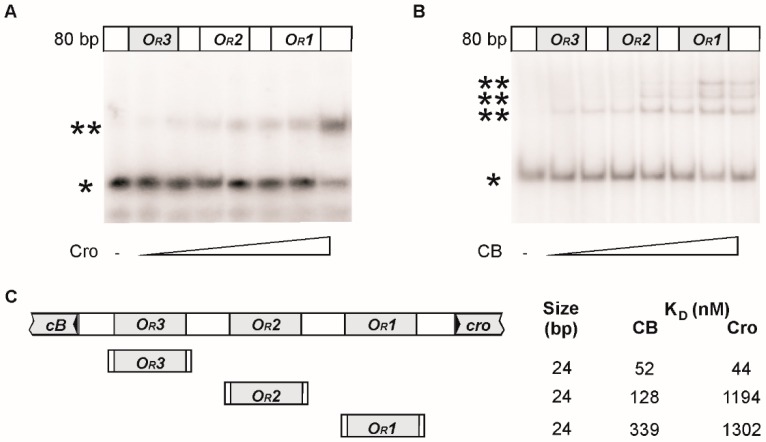
Analysis of ϕKO2 CB and Cro binding to target DNAs. Protein/DNA binding was investigated with ^32^P-5′-labelled oligonucleotides. (**A**) Cro and (**B**) CB repressor binding to a 80 bp target sequence representing the whole intergenic region (*O_R_*) of ϕKO2. The lanes show binding reactions of 1 nM target DNA and appropriate amounts of pBR329 competitor DNA that were incubated with 0, 0.01, 0.025, 0.05, 0.075, 0.1, 0.125, and 0.15 μM of the purified repressor protein. The symbols * and ** indicate free and bound DNA, respectively; (**C**) schematic illustration of target sequences that were tested to determine the apparent equilibrium dissociation constant (*K_D_*) of the ϕKO2 CB and Cro repressor/DNA complexes. *K_D_* was calculated from two independent experiments.

Using oligonucleotides as substrates the binding sites could be narrowed down to 24 bp (Figure 4). Strongest binding of CB was observed to *O_R_1*; the operators *O_R_2* and *O_R_3* were bound more weakly. Up to three protein/DNA complexes were obtained with increasing amounts of CB added to a 80 bp substrate containing the three operators indicating that all *O_R_* sites can be occupied by this protein. By contrast, only one protein/DNA complex was obtained with Cro.

### 3.3. Mutational Analysis of the Target Sites

**Figure 5:**
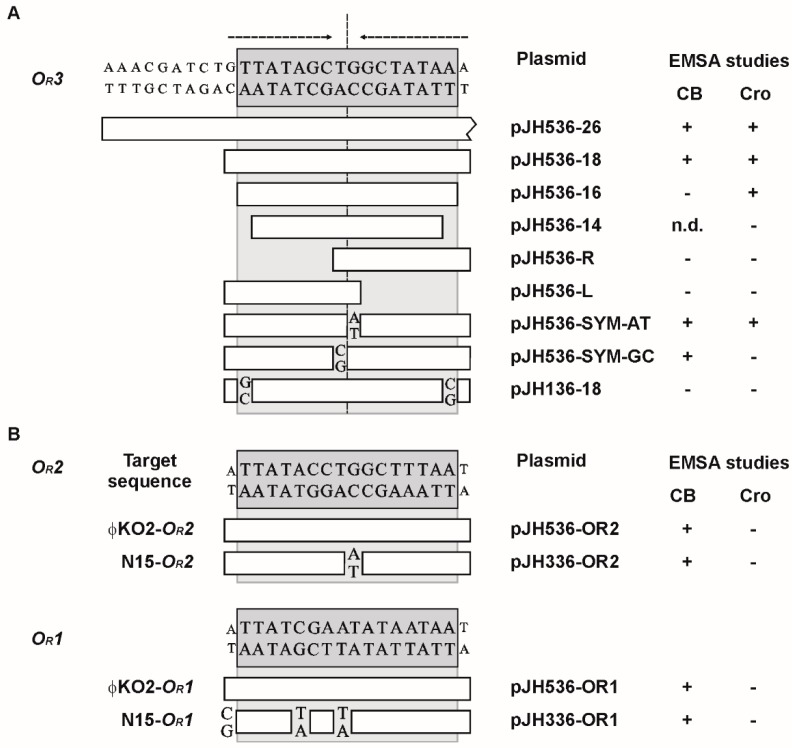
Sequence requirements and mutational analysis of the ϕKO2 repressor binding sites. Substrates used for EMSA binding assays are given. For this study recombinant plasmids containing the target sequences were used. Base pair exchanges in the target sequences are indicated. On the right the results of the repressor binding tests are presented (+, band shift; -, no band shift; n.d., not determined). (**A**) Repressor binding to the identical ϕKO2 and N15 *O_R_3* operator. The two-fold rotational symmetry of the *O_R_3* site is indicated by arrows. Substrate pJH136-18 represents the PY54 *O_R_3* operator site; (**B**) repressor binding to the ϕKO2 and N15 *O_R_2* and *O_R_1* operators.

To determine nucleotides essential for repressor binding, the sizes of the substrates were further reduced. For the CB repressor the minimal target size was 18 bp (Figure 5A). The lytic repressor Cro even bound to the 16 bp *O_R_3* site. Half sides of *O_R_3* and the related PY54 *O_R_3* site, which differs only by its peripheric nucleotides (plasmid pJH136-18), were not bound by the repressors. Thus, as with the PY54 Cro protein, the outmost nucleotides of the 16 bp operators determine the host specificity of the ϕKO2 repressors [10]. We additionally studied the importance of the central T-G nucleotides within the ϕKO2 *O_R_3* sequence. Constructs containing an A instead of the G were still bound by both CB and Cro, whereas replacement of the T by a C resulted in a loss of Cro binding. Therefore, a fully symmetrical *O_R_3* site is recognized by the ϕKO2 repressors if the center is occupied by a T-A motif (…GCT-AGC…). On the other hand, the symmetrical sequence (...GCC-GGC...) does not support binding by Cro, an effect that has already been reported for the PY54 Cro protein [10]. While the ϕKO2 *O_R_3* operator is identical to that of N15, the *O_R_2* and *O_R_1* operators of the phages show some differences (Figure 5). For that reason, we also studied binding of the ϕKO2 prophage repressor to the N15 operators *O_R_2* and *O_R_1*. Both N15 operators were bound by ϕKO2 CB confirming the rather low binding specificity of this protein (Figure 5B).

### 3.4. ϕKO2 Plasmid Prophage Replication Is Controlled by CB But Not by Cro

**Figure 6:**
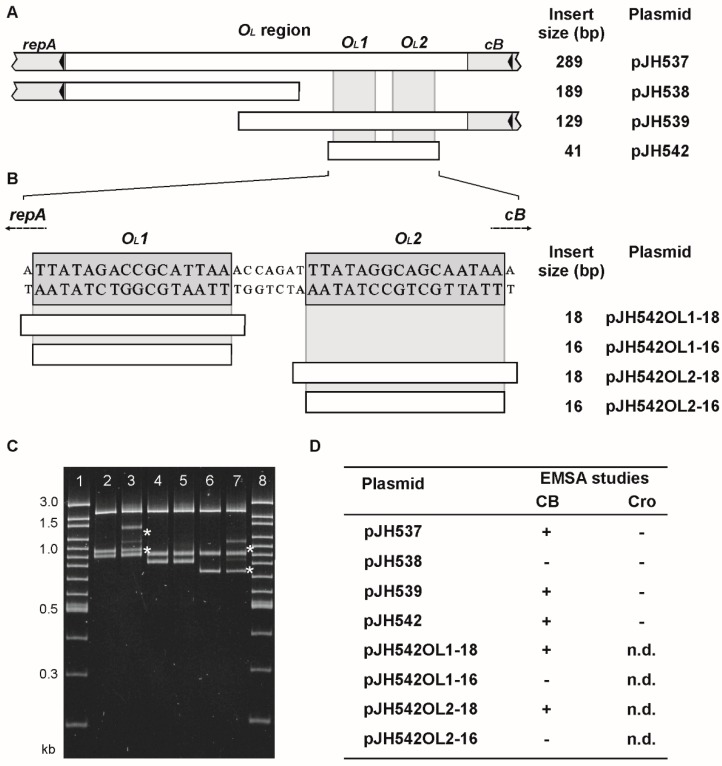
ϕKO2 repressor binding to the *O_L_* region. Binding assays were performed by EMSA using cloned target sequences. (**A**) Schematic overview of the *O_L_* region of ϕKO2. On the right, the sizes of the respective substrates are given; (**B**) the nucleotide sequences of the *O_L_1* and *O_L_2* operators are shown. Constructs containing the 18 bp or 16 bp target sites are listed on the right; (**C**) EMSA of ϕKO2 CB binding to the *O_L_* operators. Lanes 1 and 8, 100 bp plus marker ladder; lanes 2 and 3, pJH537 without and with CB (0.125 µM); lanes 4 and 5, pJH538 without and with CB (0.125 µM); lanes 6 and 7, pJH539 without and with CB (0.125 µM). Asterisks indicate complexes formed by binding of the CB repressor to the DNA targets; (**D**) summary of the results (+, band shift; -, no band shift; n.d., not determined).

Binding of the N15 prophage repressor to two *O_L_* operators upstream from the replication gene *repA* leads to a repression of *repA* transcription and a decrease of the plasmid prophage copy number [9]. Similarly, ϕKO2 mini-plasmids devoid of *cB* have a significantly higher copy number than mini-plasmids containing the prophage repressor gene (data not shown). We, thus, wanted to know whether the ϕKO2 *O_L_* region is a target for the ϕKO2 repressors. The *O_L_* region was only bound by CB, not by Cro (Figure 6). Binding exclusively occurred with the right part of the region harboring two putative operator sites (*O_L_1* and *O_L_2*). As with the *O_R_* operators the minimal size of the *O_L_* sites bound by CB were comprised of 18 bp. From the data it can be concluded that binding of the ϕKO2 prophage repressor to the *O_L_* region may interfere with *repA* transcription resulting in a reduction of the plasmid prophage copy number. On the other hand, Cro does not apparently affect plasmid replication. Whether there is a long range cooperatively between *O_R_* and *O_L_* binding of the prophage repressor, similar to that reported for lambda CI [24], is not known. We studied oligomerization of CB by glutaraldehyde crosslinking and detected some tetramers and probably also octamers (data not shown). Therefore, further experiments are necessary to ascertain whether cooperative binding of CB is involved in the regulation of plasmid prophage replication.

### 3.5. The *O_R_* Region Harbors Several Very Strong *cro* Promoters

**Figure 7:**
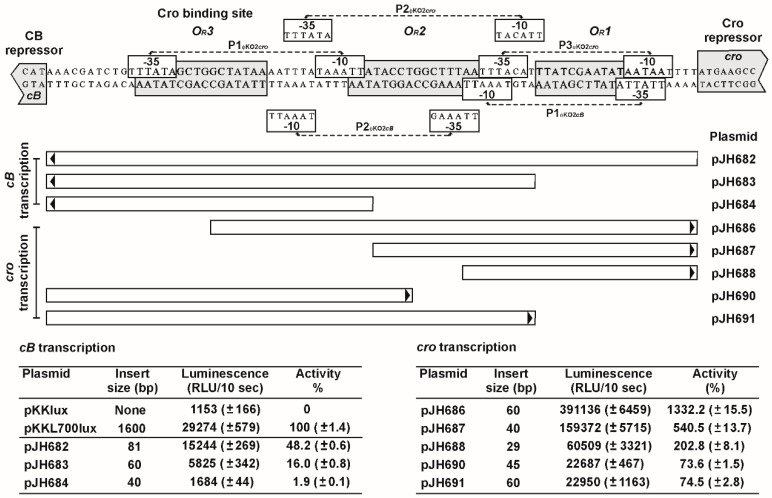
Identification of functional *cB* and *cro* promoters in ϕKO2. The upper part presents the sequence of the DNA region between the repressor genes *cB* and *cro*. Repressor binding sites and promoter sequences are boxed. Below, DNA fragments analyzed for *cB* and *cro* promoter activity are shown. Arrows indicate the direction in which the fragments were inserted into the promoter search vector pKKlux. The tables at the bottom show bioluminescence values of the constructs compared to those of pKKL700. Standard deviations are given in brackets.

**Figure 8:**
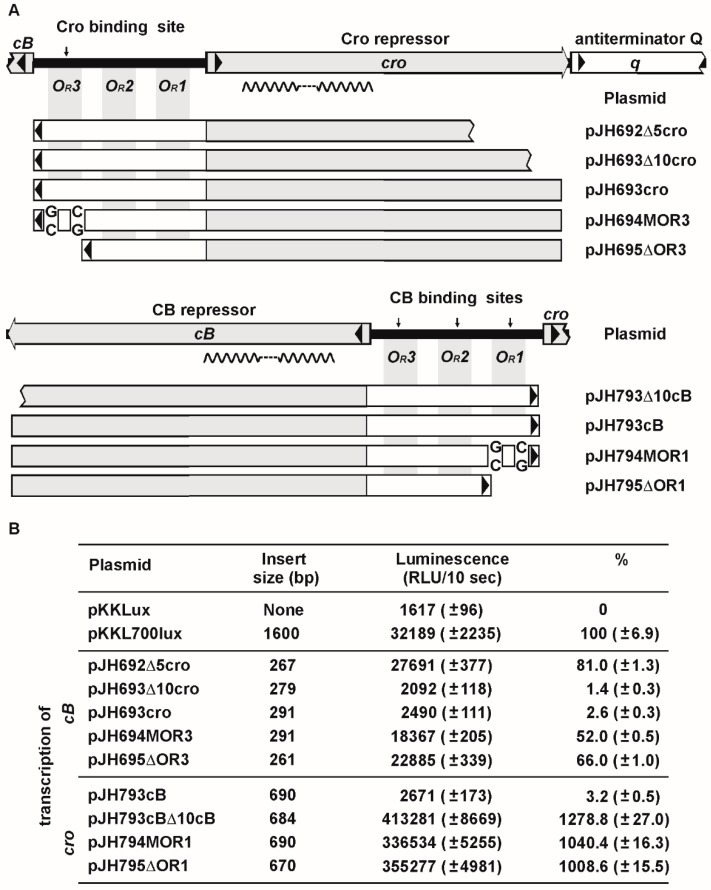
Repression of ϕKO2 *cB* and *cro* transcription by CB and Cro binding within the *O_R_* region. (**A**) Constructs tested for bioluminescence activity are illustrated. Mutations introduced in the *O_R_1* and *O_R_3* operator sites of pJH794MOR1 and pJH694MOR3, respectively, are indicated. In pJH794ΔOR1 and pJH694ΔOR3, the respective sites are lacking; (**B**) bioluminescence of the constructs compared to those of pKKL700. Standard deviations are shown in brackets.

To understand the regulation of *cB* and *cro* transcription in conjunction with repressor binding, possible promoters within the *O_R_* region were identified and analyzed. For the prophage repressor gene, two putative promoters were detected (Figure 7). Both promoters revealed moderate activity, compared to the promoter of the control plasmid pKKL700lux. The P1 promoter is located close to the *cro* start codon, P2 borders on the *O_R_3* operator. Due to the position of the *O_R_3* operator site and the promoters, it is likely that Cro binding to *O_R_3* results in a strong repression of *cB* transcription. This assumption was confirmed by reporter assays with a plasmid containing the complete *O_R_* region and *cro*. The construct (pJH693cro) showed only marginal luminescence (Figure 8) while constructs in which the *O_R_3* site was mutated (pJH694MOR3) or deleted (pJH695ΔOR3) gave significantly higher luminescence values. On the other hand, autoregulation of *cB* transcription might be more balanced since binding of the prophage repressor to the *O_R_1* operator may still allow transcription of *cB* by promoter P2.

For *cro*, three promoters overlapping with the *O_R_* operators were identified (Figure 7). All recombinant plasmids harboring one or more *cro* promoters revealed strong luminescence. By contrast, a plasmid containing *cB* in addition to the intergenic region (pJH793cB) showed almost no luciferase activity indicating that binding of the prophage repressor to the *O_R_* operators blocked *cro* transcription (Figure 8). Derivatives of this plasmid with a mutated or deleted *O_R_1* operator exhibited strong luciferase activity but from the measured luminescence values it can be inferred that binding of CB to *O_R_2* and *O_R_3* occurred to some extent since moderate repression of *cro* transcription was still detectable.

To determine the importance of the repressor proteins’ C-terminus for DNA binding, constructs containing truncated repressor genes were designed and tested by reporter assays. While a prophage repressor gene deprived of its six terminal nucleotides did not show any repression of *cro* transcription, a *cro* derivative lacking 12 nucleotides at the 3′-end repressed *cB* transcription like the wild-type gene. Further stepwise reduction, each with six nucleotides, was performed demonstrating that only a *cro* gene lacking at least 24 nucleotides did not mediate any DNA binding. Thus, the C-terminal amino acids of Cro are not essential for DNA binding, which matches the observation of Dubrava et al. who reported for the closely-related N15 Cro repressor that five to seven C-terminal residues of its A and B chain are disordered [11]. By contrast, the region towards the N-terminus of the protein forms five alpha helices that are important for the recognition and binding of the target DNA and for the dimer interface. The Cro repressors of N15 and ϕKO2 obviously belong to the same category (all-alpha helical dimer) of Cro proteins as PY54 Cro, even though they are only 31% (N15) and 35% (ϕKO2) identical to their counterpart in PY54. What these proteins have in common and what separates them from the Cro proteins of lambda and P22 is the unexpected high binding specificity for a single operator. This is particularly surprising for ϕKO2 (and probably also for N15) since these two phages possess, contrary to PY54, three operators between *cB* and *cro* and two operators between *cB* and *repA* which are all bound by the prophage repressor CB. The structural analysis of N15 Cro revealed that this protein has a fold comprising five alpha helices, which is very similar to the fold of P22 Cro [11]. There are two major differences between the backbone structures of the proteins, a diverging orientation of the fifth helix and a shorter third (“recognition”) helix in N15 Cro. Computational studies on the N15 Cro recognition helix and cognate *O_R_* consensus half-sites suggested that this repressor also binds to the five operators [13]. However, the authors point out that there is no guarantee that all of the binding sites predicted by the applied method are correct. It is conceivable that different lengths of the recognition helix of the P22 and N15 Cro proteins result in different binding specificities. On the other hand, N15 Cro dimerizes in solution much more strongly than P22 Cro and is, in this respect, similar to the otherwise unrelated Cro repressor of lambda. Although Cro proteins exhibiting strong dimerization are mostly monomeric in solution at the nanomolar concentrations typically required for operator binding [25,26,27]. A strong dimerization may increase the affinity of the free protein for operator DNA by stabilization of the functionally competent form of the protein [11]. However, this speculation contrasts with the binding properties of the telomere phage Cro proteins. Hence, it is mandatory to perform structure analyses not only with purified repressors, but also with protein/DNA complexes.

## 4. Conclusions

The data of our study demonstrate that the *immB* region of phage ϕKO2, and probably also that of the related phage N15, is reminiscent of the genetic switch of lambda-like phages, but functions in a different way. As already shown for the N15 prophage repressor, CB of ϕKO2 binds to five operator sites with varying affinity. By doing this, it regulates the transcription of *cro*, *repA* and of its own gene. Cro, however, exclusively binds to the *O_R_3* operator upstream from *cB*. It can repress the transcription of the prophage repressor gene, but it apparently neither regulates its own synthesis, nor the plasmid copy number. PY54 Cro binds similarly [10] and presumably also the lytic repressor of N15. Hence, the three enterobacterial telomere phages share the same principle of Cro-mediated gene regulation and differ in this respect from other lambda-like phages. We will now analyze binding of the PY54 prophage repressor since this phage contains only one operator site (*O_R_3*) between *cI* and *cro* and it will be interesting to see how CI regulates lysogeny. Preliminary data [8,10] suggest that the genetic switch of PY54 and also those of marine telomere phages are individually different from the N15/ϕKO2 switch, which underlines the diversity of this regulatory system in telomere phages.

## Supplementary Materials

The following are available online at www.mdpi.com/1999-4915/8/8/213/s1, Figure S1: Organization of the immunity region and the operator sites (*O_L_* and *O_R_*) of lambda, ϕKO2 and PY54, Table S1: Bacterial strains and plasmids.
