# Supplementary material for: Binding Specificities of the Telomere Phage ϕKO2 Prophage Repressor CB and Lytic Repressor Cro
Source: Viruses. 2016 Aug 3;8(8):213. doi: 10.3390/v8080213 (PMC4997575; doi:10.3390/v8080213)
Supplement: Supplementary file 1 [file viruses-08-00213-s001.pdf]

Supplementary Materials: Binding Specificities of the Telomere Phage φKO2 Prophage Repressor CB and Lytic Repressor Cro

Jens Andre Hammerl [1], Claudia Jäckel [1], Erich Lanka [2], Nicole Roschanski [3] and Stefan Hertwig [1,*]

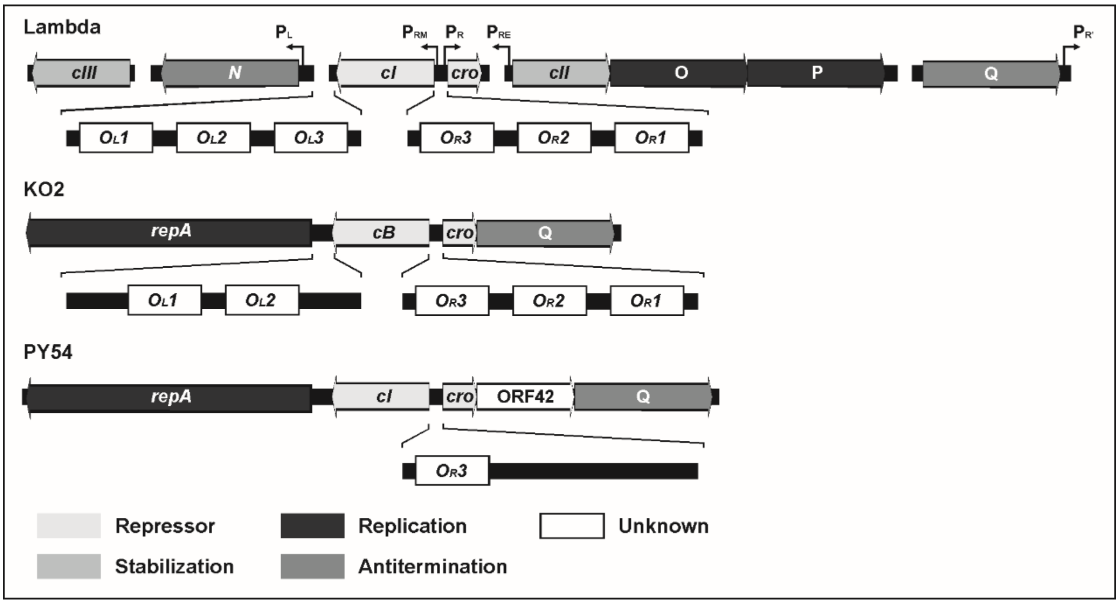

Figure S1. Organization of the immunity region and the operator sites (O_L and O_R) of lambda, φKO2 and PY54.

Table S1. Bacterial strains and plasmids.

Strains	Description	Reference or Origin
E. coli SCS1	*recA1 endA1 gyrA96 thi-1 hsdR17*(r_K-m_K+) *supE44 relA1*	Stratagene
E. coli UT5600	F^- *ara-14 leuB6 secA6 lacY1 proC14 tsx-67* φ (*ompT-fepC*)266 *entA403 trpE38 rfbD1 rpsL109 xyl-5 mtl-1 thi-1*	NEB
E. coli Genehogs	F^- *mcrA* Δ(*mrr-hsdRMS-mcrBC*) φ80 *lacZ* ΔM15 Δ*lacX74 recA1 araD139* Δ(*ara-leu*)7697 *galU galK rpsL* (Sm^r) *endA1 nupG fhuA*::IS2 (confers phage T1 resistance)	Invitrogen
K. oxytoca CCUG 15788	wild type strain, isolated from a mineral oil emulsion tank in Göteborg, Sweden; contains the φKO2 prophage	[1]
**Plasmids**	**Description**	**Reference or Origin**
pBR329	4.2 kb cloning vector, pMB1 replicon; Ap^r, Cm^r, Tc^r	[2]
pMS470Δ8cat	6.9 kb cloning vector, pMB1 replicon, P_{tac}/ *lacI*; T7 gene Shine-Dalgarno sequence; Ap^r, Cm^r	[3]
pKKlux	7.7 kb promoter probe vector; pMB1 replicon; promoterless *luxAB* genes from *Vibrio harveyi*; T_1T_2 of *rrnB*; Ap^r	[4]
pKKL700lux	9.3 kb; derived from pKKlux, contains the *Solanum tuberosum* ST-LS promoter; Ap^r	[4]
pJH531	pBR329 Δ[BamHI-HindIII] Ω[φKO2 PCR, φKO2 39.098-39.259, BamHI-HindIII]	Figure 3, this work
pJH532	pBR329 Δ[BamHI-HindIII] Ω[φKO2 PCR, φKO2 39.179-39.259, BamHI-HindIII]	Figure 3, this work
pJH533	pBR329 Δ[BamHI-HindIII] Ω[φKO2 PCR, φKO2 39.098-39.198, BamHI-HindIII]	Figure 3, this work
pJH534	pBR329 Δ[BamHI-HindIII] Ω[φKO2 PCR, φKO2 39.206-39.259, BamHI-HindIII]	Figure 3, this work
pJH535	pBR329 Δ[BamHI-HindIII] Ω[φKO2 PCR, φKO2 39.195-39.244, BamHI-HindIII]	Figure 3, this work
pJH536	pBR329 Δ[BamHI-HindIII] Ω[φKO2 PCR, φKO2 39.098-39.175, BamHI-HindIII]	Figure 3, this work
pJH536Δ*cI*	pBR329 Δ[BamHI-HindIII] Ω[φKO2 PCR, φKO2 39.146-39.175, BamHI-HindIII]	Figure 3, this work
pJH536-26	pBR329 Δ[BamHI-HindIII] Ω[φKO2 PCR, φKO2 39.148-39.173, BamHI-HindIII]	Figure 5, this work
pJH536-18	pBR329 Δ[BamHI-HindIII] Ω[φKO2 OR3, φKO2 39.156-39.173, BamHI-HindIII]	Figure 5, this work
pJH536-16	pBR329 Δ[BamHI-HindIII] Ω[φKO2 OR3-2, φKO2 39.157-39.172, BamHI-HindIII]	Figure 5, this work
pJH536-14	pBR329 Δ[BamHI-HindIII] Ω[φKO2 OR3-4, φKO2 39.158-39.171, BamHI-HindIII]	Figure 5, this work
pJH536-R	pBR329 Δ[BamHI-HindIII] Ω[φKO2 OR3-R, , φKO2 39.162-39.171, BamHI-HindIII]	Figure 5, this work
pJH536-L	pBR329 Δ[BamHI-HindIII] Ω[φKO2 OR3-L, , φKO2 39.158-39.167, BamHI-HindIII]	Figure 5, this work
pJH536-SYM-AT	pBR329 Δ[BamHI-HindIII] Ω[φKO2 OR3AT, φKO2 39.156-39.173, BamHI-HindIII]	Figure 5, this work
pJH536-SYM-GC	pBR329 Δ[BamHI-HindIII] Ω[φKO2 OR3GC, φKO2 39.156-39.173, BamHI-HindIII]	Figure 5, this work
pJH136-18	pBR329 Δ[BamHI-HindIII] Ω[PY54 OR3, PY54 32.536-32.551, BamHI-HindIII]	Figure 5
φKO2-OR2	pBR329 Δ[BamHI-HindIII] Ω[φKO2 OR2, φKO2 39.182-39.200, BamHI-HindIII]	Figure 5, this work
N15-OR2	pBR329 Δ[BamHI-HindIII] Ω[N15 OR2, N15 34.754-34.771, BamHI-HindIII]	Figure 5, this work
φKO2-OR1	pBR329 Δ[BamHI-HindIII] Ω[φKO2 OR1, φKO2 39.205-39.223, BamHI-HindIII]	Figure 5, this work
N15-OR1	pBR329 Δ[BamHI-HindIII] Ω[N15 OR1, N15 34.777-34791, BamHI-HindIII]	Figure 5, this work
pJH537	pBR329 Δ[BamHI-HindIII] Ω[φKO2 OR2, φKO2 38.275-38.563, BamHI-HindIII]	Figure 6, this work

pJH538	pBR329 Δ[BamHI-HindIII] Ω[φKO2 OR2, φKO2 38.275-38.463, BamHI-HindIII]	Figure 6, this work
pJH539	pBR329 Δ[BamHI-HindIII] Ω[φKO2 OR2, φKO2 38.435-38.563, BamHI-HindIII]	Figure 6, this work
pJH542	pBR329 Δ[BamHI-HindIII] Ω[φKO2 OR2, φKO2 38.483-38.523, BamHI-HindIII]	Figure 6, this work
pJH542OL1-18	pBR329 Δ[BamHI-HindIII] Ω[φKO2 OR2, φKO2 38.483-38.500, BamHI-HindIII]	Figure 6, this work
pJH542OL1-16	pBR329 Δ[BamHI-HindIII] Ω[φKO2 OR2, φKO2 38.484-38.499, BamHI-HindIII]	Figure 6, this work
pJH542OL2-18	pBR329 Δ[BamHI-HindIII] Ω[φKO2 OR2, φKO2 38.506-38.523, BamHI-HindIII]	Figure 6, this work
pJH542OL2-16	pBR329 Δ[BamHI-HindIII] Ω[φKO2 OR2, φKO2 38.507-38.522, BamHI-HindIII]	Figure 6, this work
pJH541-2	pMS470Δ8cat Δ[NdeI-HindIII] Ω[φKO2 PCR, *cB* φKO2 38.534-39.145, NdeI-HindIII]	This work
pJH542-2	pMS470Δ8cat Δ[NdeI-HindIII] Ω[φKO2 PCR, *cro* φKO2 39.226-39.438, NdeI-HindIII]	This work
pJH682	pKKluxBamHI Δ[SmaI-XbaI] Ω[φKO2 PCR, φKO2 39.146-39.225, SmaI-XbaI]	Figure 7, this work
pJH683	pKKluxBamHI Δ[SmaI-XbaI] Ω[φKO2 PCR, φKO2 39.146-39.205, SmaI-XbaI]	Figure 7, this work
pJH684	pKKluxBamHI Δ[SmaI-XbaI] Ω[φKO2 PCR, φKO2 39.146-39.185, SmaI-XbaI]	Figure 7, this work
pJH686	pKKluxBamHI Δ[SmaI-XbaI] Ω[φKO2 PCR, φKO2 39.166-39.225, SmaI-XbaI]	Figure 7, this work
pJH687	pKKluxBamHI Δ[SmaI-XbaI] Ω[φKO2 PCR, φKO2 39.186-39.225, SmaI-XbaI]	Figure 7, this work
pJH688	pKKluxBamHI Δ[SmaI-XbaI] Ω[φKO2 PCR, φKO2 39.197-39.225, SmaI-XbaI]	Figure 7, this work
pJH690	pKKluxBamHI Δ[SmaI-XbaI] Ω[φKO2 PCR, φKO2 39.146-39.190, SmaI-XbaI]	Figure 7, this work
pJH691	pKKluxBamHI Δ[SmaI-XbaI] Ω[φKO2 PCR, φKO2 39.146-39.205, SmaI-XbaI]	Figure 7, this work
pJH692cro	pKKluxBamHI Δ[SmaI-XbaI] Ω[φKO2 PCR, φKO2 39.148-39.414, SmaI-XbaI]	Figure 8, this work
pJH693croΔ10cro	pKKluxBamHI Δ[SmaI-XbaI] Ω[φKO2 PCR, φKO2 39.148-39.427, SmaI-XbaI]	Figure 8, this work
pJH693cro	pKKluxBamHI Δ[SmaI-XbaI] Ω[φKO2 PCR, φKO2 39.148-39.438, SmaI-XbaI]	Figure 8, this work
pJH694MOR3	pKKluxBamHI Δ[SmaI-XbaI] Ω[φKO2 PCR, φKO2 39.148-39.438, SmaI-XbaI]	Figure 8, this work
pJH695ΔOR3	pKKluxBamHI Δ[SmaI-XbaI] Ω[φKO2 PCR, φKO2 39.178-39.438, SmaI-XbaI]	Figure 8, this work
pJH793cB	pKKluxBamHI Δ[SmaI-XbaI] Ω[φKO2 PCR, φKO2 38.534-39.223, SmaI-XbaI]	Figure 8, this work
pJH793cBΔ10cB	pKKluxBamHI Δ[SmaI-XbaI] Ω[φKO2 PCR, φKO2 38.540-39.223, SmaI-XbaI]	Figure 8, this work
pJH794MOR1	pKKluxBamHI Δ[SmaI-XbaI] Ω[φKO2 PCR, φKO2 38.534-39.223, SmaI-XbaI]	Figure 8, this work
pJH795Δ5OR1	pKKluxBamHI Δ[SmaI-XbaI] Ω[φKO2 PCR, φKO2 38.534-39.203, SmaI-XbaI]	Figure 8, this work

References

1. Casjens, S.R.; Gilcrease, E.B.; Huang, W.M.; Bunny, K.L.; Pedulla, M.L.; Ford, M.E.; Houtz, J.M.; Hatfull, G.F.; Hendrix, R.W. The pKO2 linear plasmid prophage of *Klebsiella oxytoca*. *J. Bacteriol.* **2004**, *186*, 1818–1832.
2. Covarrubias, L.; Bolivar, F. Construction and characterization of new cloning vehicles. VI. Plasmid pBR329, a new derivative of pBR328 lacking the 482-base-pair inverted duplication. *Gene* **1982**, *17*, 79–89.
3. Balzer, D.; Ziegelin, G.; Pansegrau, W.; Kruft, V.; Lanka, E. KorB protein of promiscuous plasmid RP4 recognizes inverted sequence repetitions in regions essential for conjugative plasmid transfer. *Nucleic Acids Res.* **1992**, *20*, 1851–1858.
4. Jacob, D.; Lewin, A.; Meister, B.; Appel, B. Plant-specific promoter sequences carry elements that are recognised by the eubacterial transcription machinery. *Transgenic Res.* **2002**, *11*, 291–303.
